# Cardiolipin metabolism regulates expression of muscle transcription factor MyoD1 and muscle development

**DOI:** 10.1016/j.jbc.2023.102978

**Published:** 2023-02-04

**Authors:** Linh Vo, Michael W. Schmidtke, Nevton T. Da Rosa-Junior, Mindong Ren, Michael Schlame, Miriam L. Greenberg

**Affiliations:** 1 Department of Biological Sciences, Wayne State University, Detroit, Michigan, USA; 2 Department of Anesthesiology, Perioperative Care, and Pain Medicine at New York University Grossman School of Medicine, New York, New York, USA; 3 Department of Cell Biology at New York University Grossman School of Medicine, New York, New York, USA

**Keywords:** Barth syndrome, cardiolipin, TAFAZZIN, MyoD1, skeletal muscle development, myogenesis, BTHS, Barth syndrome, CL, cardiolipin, EV, empty vector, MHC, myosin heavy chain, MKX, Mohawk, MyoD1, myoblast determination protein 1, PANTHER, Protein ANalysis THrough Evolutionary Relationships, qPCR, quantitative PCR, RNA-seq, RNA sequencing, TAZ-KO, TAFAZZIN-knockout, Wls, Wntless

## Abstract

The mitochondrial phospholipid cardiolipin (CL) is critical for numerous essential biological processes, including mitochondrial dynamics and energy metabolism. Mutations in the CL remodeling enzyme TAFAZZIN cause Barth syndrome, a life-threatening genetic disorder that results in severe physiological defects, including cardiomyopathy, skeletal myopathy, and neutropenia. To study the molecular mechanisms whereby CL deficiency leads to skeletal myopathy, we carried out transcriptomic analysis of the TAFAZZIN-knockout (TAZ-KO) mouse myoblast C2C12 cell line. Our data indicated that cardiac and muscle development pathways are highly decreased in TAZ-KO cells, consistent with a previous report of defective myogenesis in this cell line. Interestingly, the muscle transcription factor myoblast determination protein 1 (MyoD1) is significantly repressed in TAZ-KO cells and TAZ-KO mouse hearts. Exogenous expression of MyoD1 rescued the myogenesis defects previously observed in TAZ-KO cells. Our data suggest that MyoD1 repression is caused by upregulation of the MyoD1 negative regulator, homeobox protein Mohawk, and decreased Wnt signaling. Our findings reveal, for the first time, that CL metabolism regulates muscle differentiation through MyoD1 and identify the mechanism whereby MyoD1 is repressed in CL-deficient cells.

Barth syndrome (BTHS) is a rare genetic disorder caused by mutations in tafazzin (TAFAZZIN), a protein that remodels nascent cardiolipin (CL) by incorporating more unsaturated acyl chains (1, 2). CL is the signature phospholipid of the mitochondrial inner membrane and plays important roles in essential biological processes including cellular homeostasis, energy metabolism, and mitochondrial dynamics (3, 4, 5, 6). BTHS patients suffer from a broad range of defects, including cardiomyopathy and skeletal myopathy, exercise intolerance, and neutropenia (7, 8). Although most BTHS symptoms are indicative of mitochondrial dysfunction, the molecular mechanisms linking CL deficiency to BTHS pathology are unknown. Currently there is no cure for BTHS, and interventions are instead aimed at alleviating symptoms to increase quality of life and longevity of patients.

One of the major clinical manifestations of BTHS is skeletal muscle myopathy, characterized by muscle weakness and wasting, exercise intolerance, and hypotonia (9). Skeletal myopathy not only impacts muscle capabilities during exercise but also poses a significant burden for completing day-to-day activities and can progress to the point of requiring ambulatory assistance (10). The mechanisms that link TAFAZZIN mutations to skeletal myopathy are poorly understood, and this knowledge gap precludes the development of effective treatments for BTHS patients.

Muscle differentiation, or myogenesis, is the process of forming muscle tissue through the fusion of myoblasts into multinucleated myotube fibers during embryogenesis and muscle regeneration (11). Myogenesis is a complex process tightly controlled by myogenic regulatory factors, a family of transcription factors that include myoblast determination protein 1 (MyoD1). MyoD1 is a master regulator and co-activator of several downstream genes responsible for myogenic differentiation (12, 13). One of the most important functions of MyoD1 is to prevent myoblasts from entering the S phase of the cell cycle, a mechanism to ensure their commitment to the differentiation program (14, 15, 16, 17).

To study the defects in BTHS muscle cells, we previously constructed a TAFAZZIN-knockout (TAZ-KO) mouse myoblast C2C12 cell line using CRISPR/Cas9 (18, 19). Using this model, Lou *et al.* found that differentiation of TAZ-KO myoblasts is significantly reduced in response to differentiation induction (19). However, the mechanism whereby loss of TAFAZZIN leads to defective myogenesis is not known. In the current study, we reveal that cardiac and skeletal muscle development pathways are highly downregulated in TAZ-KO myoblasts and that the “master myogenic switch” MyoD1 is significantly decreased in these cells. We further show that the MyoD1 negative regulator homeobox protein Mohawk (MKX) is upregulated, which likely results from decreased Wnt signaling in the absence of TAFAZZIN. Our findings indicate a new regulatory role for CL in muscle development and potentially identify a new treatment strategy for BTHS skeletal myopathy based on *MyoD1*-targeted therapy.

## Results

### RNA sequencing identifies decreased muscle differentiation pathways in TAZ-KO cells

**Figure 1:**
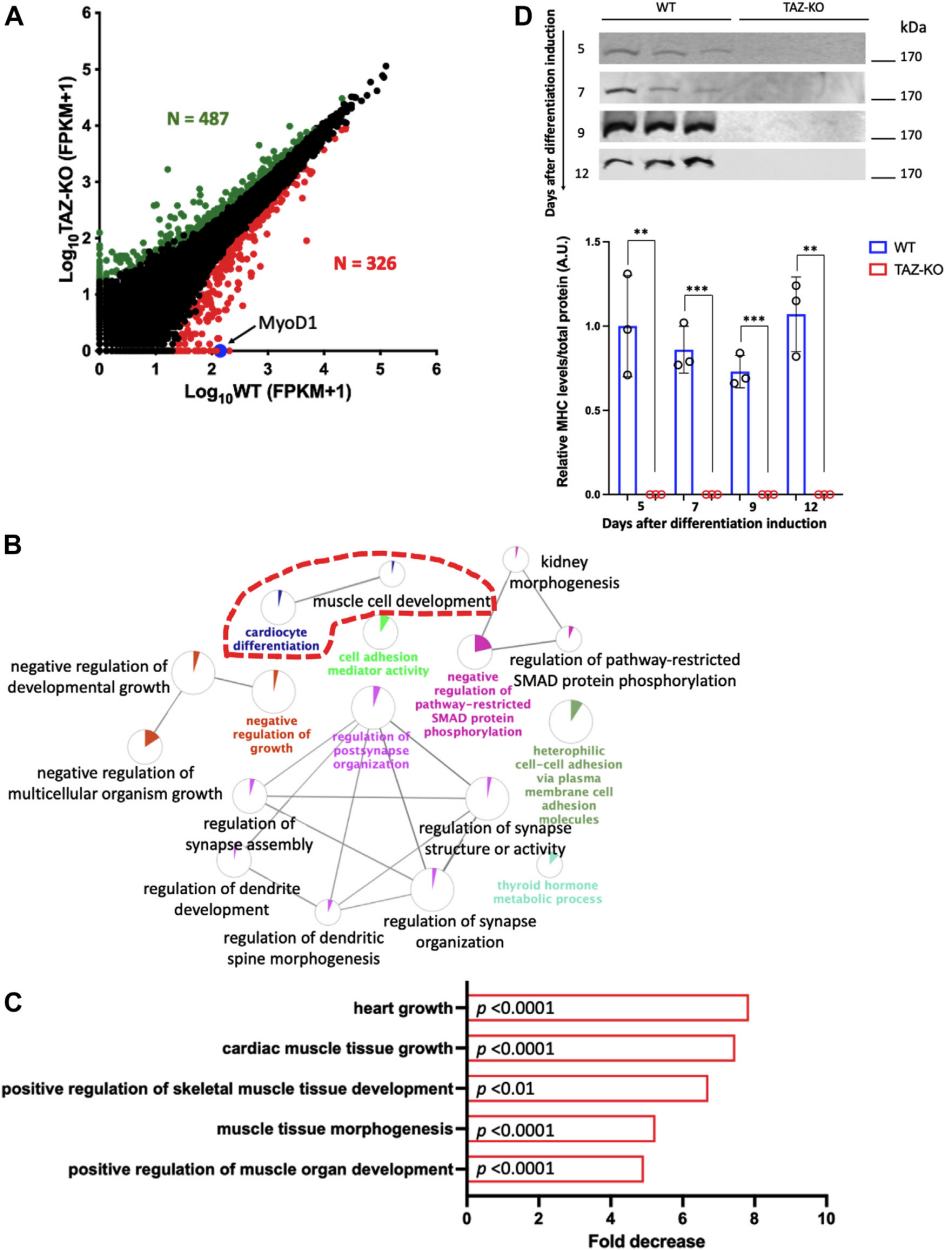
**Muscle differentiation pathways are decreased in TAZ-KO myoblasts**. *A*, scatter plot indicating changes in gene expression between undifferentiated TAZ-KO myoblasts and WT controls. Results are the average of three biological replicates. Genes with a fold change >1.5 and FDR < 0.05 were considered significant. Of 21,516 analyzed genes, 326 were downregulated (*red dots*) and 487 were upregulated (*green dots*). MyoD1 is one of the top downregulated genes in TAZ-KO cells (*blue dot* indicated by *black arrow*). *B*, gene ontology analysis of the top 100 downregulated genes based on their fold change in TAZ-KO myoblasts (*versus* WT controls) determined by RNA-seq. ClueGO (20) was used to identify significantly altered biological processes (*p* < 0.01). Cardiocyte differentiation and muscle cell development are among the most significantly suppressed pathways in TAZ-KO cells. *C*, representative decreased pathways in TAZ-KO myoblasts predicted by PANTHER pathway analysis based on RNA-seq data (22, 23). Pathways with a fold change >2 and *p* <0.05 were considered significant. The *p*-value for each pathway is shown on the respective bar. *D*, immunoblot images of MHC protein in WT and TAZ-KO cells after 5, 7, 9, and 12 days following induction of differentiation (upper panel), and their respective quantification (lower panel). MHC is not detectable in TAZ-KO cells, indicating severe myogenesis defects. All data shown are mean ± S.D. (∗∗) *p* < 0.01, (∗∗∗) *p* < 0.001. MHC, myosin heavy chain; MyoD1, myoblast determination protein 1; RNA-seq, RNA sequencing; TAZ-KO, TAFAZZIN-knockout.

In our previous study, Lou *et al.* showed that myogenic differentiation of TAZ-KO myoblasts was significantly reduced in response to serum starvation (19). To gain insight into pathways relevant to myoblast differentiation that may be altered in TAFAZZIN-deficient cells, we carried out an RNA sequencing (RNA-seq) analysis of undifferentiated TAZ-KO cells compared to wildtype (WT) C2C12 control cells. Genes differentially expressed with a fold change of >1.5 and false discovery rate <0.05 were considered significant. Of 21,516 genes that were analyzed, 487 genes were significantly upregulated and 326 genes were significantly downregulated (Fig. 1*A*). Complete expression data of all genes with their log_2_FC, logCPM, *p*-value, and false discovery rate can be found in Table S1.

ClueGO (gene ontology) analysis (20) of significantly downregulated genes in TAZ-KO myoblasts revealed that GO terms related to muscle tissue growth and development, synapse regeneration, and several metabolic processes (metabolism of reactive oxygen species, cholesterol, sterol, phosphate, and proteins) were highly decreased. Significantly upregulated genes in TAZ-KO cells were those involved in apoptotic signaling pathways and programmed cell death. Interestingly, these data are consistent with a recently published transcriptomic analysis of TAFAZZIN-deficient neutrophil progenitors, wherein genes associated with endoplasmic reticulum stress–induced apoptosis were highly increased (21).

Further analysis of the top 100 downregulated genes based on their fold change revealed that one of the most significantly suppressed pathways in TAZ-KO cells is that of cardiac muscle growth and development (Fig. 1*B*). Table 1 lists representative decreased pathways in this category and the corresponding downregulated genes. PANTHER (Protein ANalysis THrough Evolutionary Relationships) pathway analysis (22, 23) further showed that biological processes responsible for cardiac and skeletal muscle tissue development are highly decreased (5- to 8-fold) in TAZ-KO cells (Fig. 1*C*). A complete list of altered biological processes analyzed by PANTHER is shown in Table S2.

**Table 1:** Representative pathways and genes downregulated in TAZ-KO myoblasts

Representative biological pathway	Downregulated genes
Muscle cell development	*Krt19, Cxadr, Pdlim5, Uchl1, Acta1, Nexn, Ank2,****Myod1***, *Ttn, Nfatc3, Sorbs2, Foxp1*
Cardiocyte differentiation	*Cxadr, Pdlim5, Nexn,****Myod1****, Acadm, Rxra, Grem1, Ttn, Tenm4, Sorbs2, Foxp1*
Muscle cell differentiation	*Sox9, Krt19, Tbx1, Cxadr, Igfbp5, Pdlim5, Uchl1, Acta1, Nexn, Npnt, Ank2,****Myod1****, Acadm, Rxra, Ttn, Nfatc3, Sorbs2, Foxp1*
Regulation of muscle tissue development	*G6pdx, Tbx1, Edn1, Hmgcr, Cxadr, Igfbp5 Shox2, Slc25a4, Zfpm2, Nog,****Myod1****, Fdps, Fgfr1, Grem1, Bcl2, Tsc22d3, Jarid2, Foxp1*
Cardiac muscle tissue development	*Cxadr, Pdlim5, Egln1, Nexn, S1pr1, Zfpm2, Foxc1, Foxc2, Nog,****Myod1****, Acadm, Rxra, Ttn, Tenm4, Xirp2, Sorbs2, Foxp1*

Significantly decreased pathways (*p* < 0.05 by ClueGO analysis (20)) in TAZ-KO cells and downregulated genes within each pathway are shown. *Myod1* (bold) is involved in several muscle development pathways. Data were obtained from RNA-seq analysis of TAZ-KO myoblasts compared to WT controls.

**Supplemental Figure 1:**
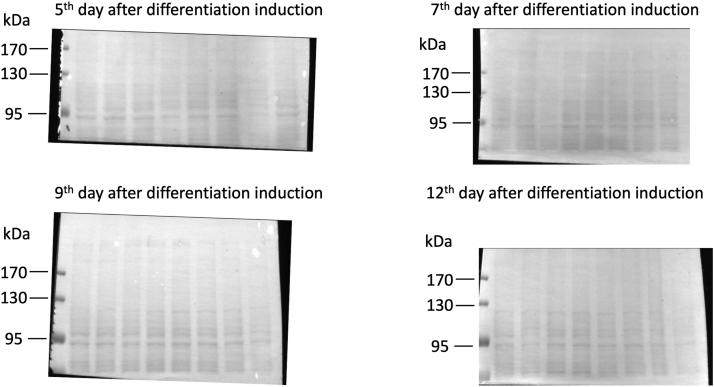


To determine whether myogenic differentiation is truly inhibited or only delayed in myoblasts lacking TAFAZZIN, we induced myoblast differentiation by culturing cells in low serum medium for an extended period of 5 to 12 days (*versus* the endpoint of 7 days by (19)). After differentiation induction, cells were collected for immunoblotting against the muscle contractile protein myosin heavy chain (MHC). As shown in Figure 1*D*, TAZ-KO cells exhibited severe inhibition of myogenic differentiation, as indicated by the total absence of detectable MHC expression. Total protein staining used for normalization of MHC can be found in Fig. S1. This suggests that loss of TAFAZZIN significantly suppresses myogenesis. The fact that TAZ-KO cells did not express any detectable MHC after 12 days of differentiation induction suggests that the myogenesis defects are severe and not simply a delay in differentiation onset. This is consistent with the repression of muscle development pathways observed in our RNA-seq analysis (Fig. 1, *B* and *C*).

### MyoD1 is highly repressed in BTHS models

**Figure 2:**
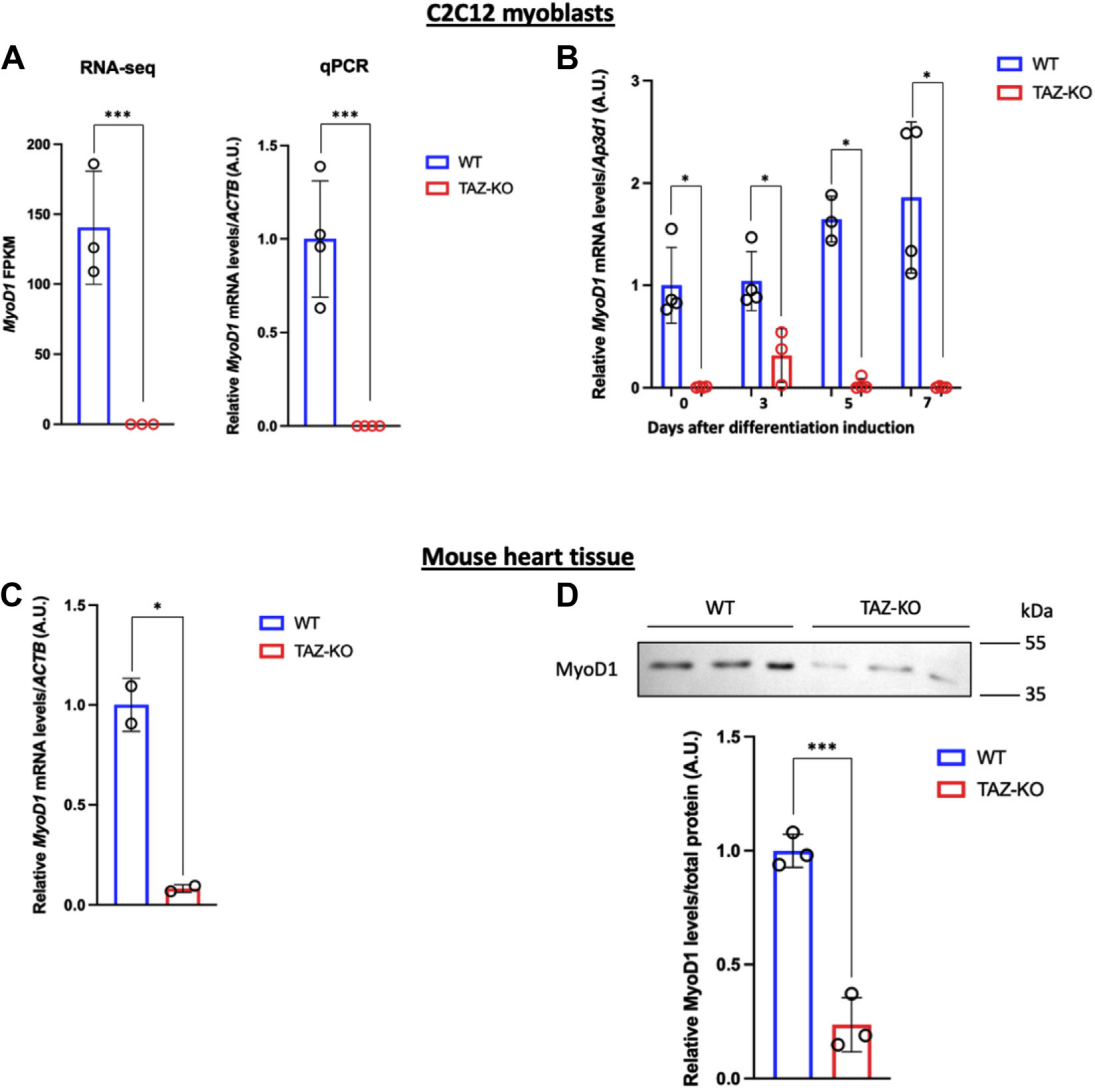
**MyoD1 is downregulated in TAZ-KO myoblasts and TAZ-KO mouse hearts.***A*, fragments per kilobase million (FPKM) values determined by RNA-seq (*left*) and qPCR analysis of *MyoD1* mRNA levels (*right*) in WT and TAZ-KO myoblasts. *β-actin* (*ACTB*) was used as an internal control for qPCR. *B*, qPCR analysis of *MyoD1* mRNA levels before and after differentiation induction. *Adapter-relatedprotein complex3subunitdelta 1* (*Ap3d1*) was used as an internal control. *C*, qPCR analysis of *MyoD1* mRNA levels in WT and TAZ-KO mouse hearts. *ACTB* was used as an internal control. *D*, immunoblot images of MyoD1 protein in WT and TAZ-KO mouse hearts (*upper panel*), and their respective quantification (*lower panel*). All data shown are mean ± S.D. ∗*p* < 0.05, ∗∗∗*p* < 0.001. MyoD1, myoblast determination protein 1; qPCR, quantitative PCR; RNA-seq, RNA sequencing; TAZ-KO, TAFAZZIN-knockout.

MyoD1 is a master transcription factor that regulates muscle tissue development and homeostasis (12, 24). Our RNA-seq data revealed that MyoD1 is one of the most downregulated genes in TAZ-KO cells (Fig. 1*A*, blue dot). This was confirmed by quantitative PCR (qPCR) analysis showing that *MyoD1* mRNA levels were borderline undetectable in both undifferentiated TAZ-KO myoblasts (Fig. 2*A*, right) and differentiation-induced TAZ-KO cells before and after induction (Fig. 2*B*). These data suggest that loss of TAFAZZIN results in long-term inhibition of MyoD1, which may be responsible for the myogenesis defects observed in TAZ-KO myoblasts.

**Supplemental Figure 2:**
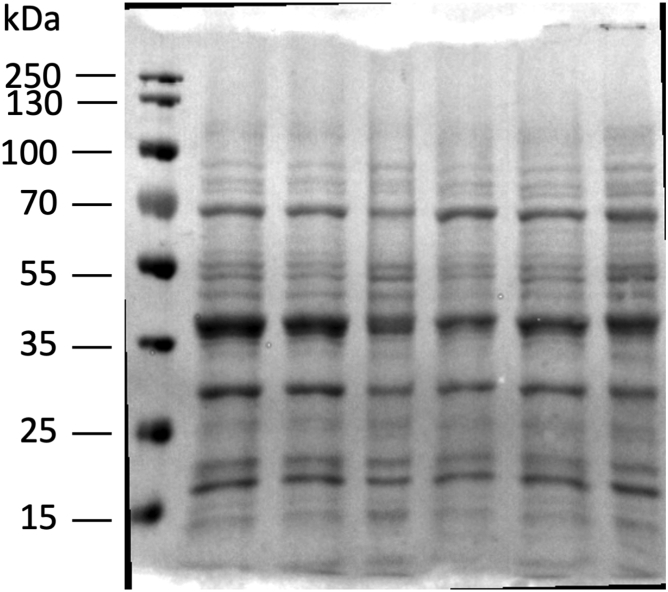


To determine if MyoD1 is also downregulated in the BTHS mouse model, we performed qPCR and immunoblotting analysis of MyoD1 in heart tissues collected from three TAZ-KO mice and three WT controls. Strikingly, we found that both MyoD1 mRNA and protein levels were significantly decreased in TAZ-KO mouse hearts compared to WT hearts (Figs. 2, *C* and *D* and S2). These data suggest that MyoD1 inhibition caused by loss of TAFAZZIN is conserved in both the *in vitro* TAZ-KO C2C12 BTHS model and *in vivo* BTHS mouse hearts.

### Exogenous MyoD1 rescues myogenesis defects caused by loss of TAFAZZIN

To determine if defective myogenesis is caused specifically by MyoD1 inhibition in TAZ-KO cells, we tested whether exogenous MyoD1 can rescue MHC levels following differentiation induction. To achieve this, we transfected TAZ-KO cells with either an empty vector (EV) or CMV-MyoD expression plasmid containing the mouse MyoD1 sequence, then measured protein expression at 5 and 7 days following differentiation induction.

**Figure 3:**
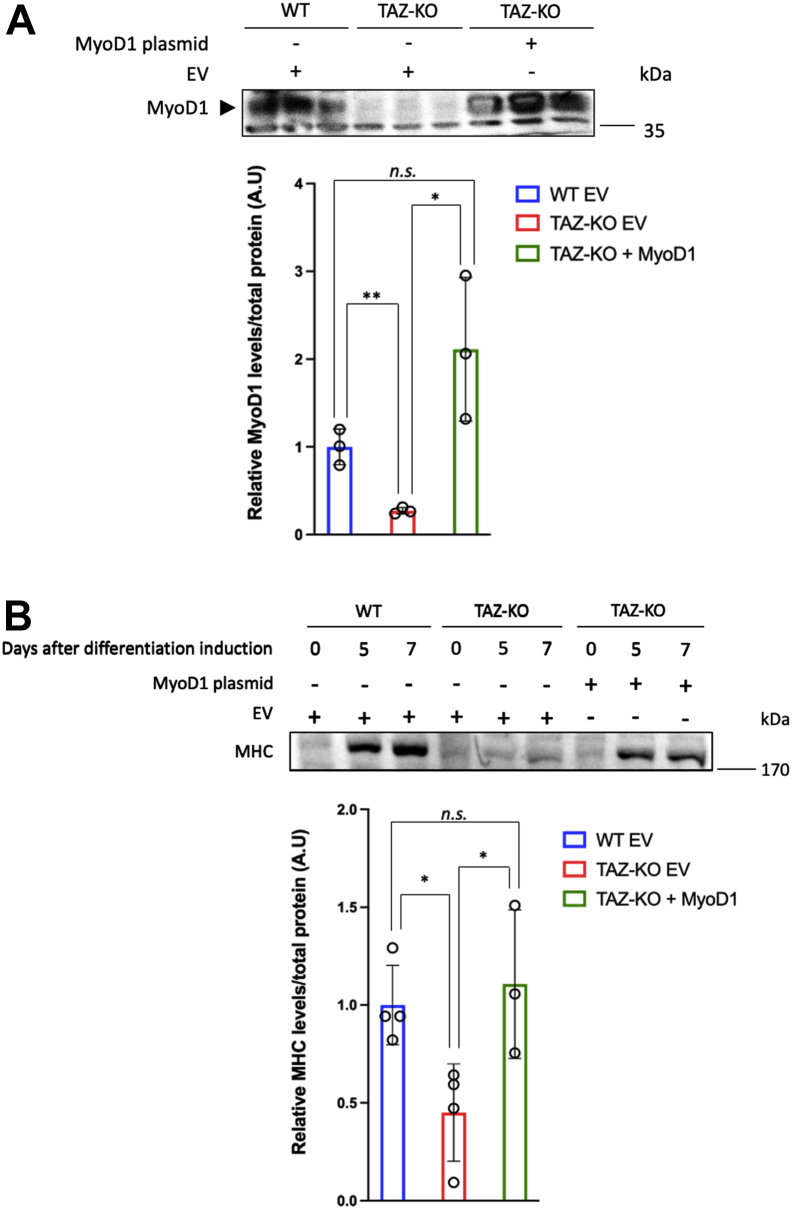
**Exogenous MyoD1 rescues the myogenesis defects of TAZ-KO cells**. *A*, MyoD1 protein is not detectable in TAZ-KO cells but can be restored by exogenous expression. Upper panel, WT, and TAZ-KO myoblasts were transfected with an empty vector (EV) (61) or CMV-MyoD plasmid (60), and after 7 days, cells were harvested for immunoblotting analysis against MyoD1. Lower panel, quantification of MyoD1 protein levels as previously described. *B*, MHC protein levels are rescued by exogenous expression of MyoD1 in TAZ-KO cells. Upper panel, cells were induced to differentiate by culturing in differentiation medium for 5 or 7 days, then harvested for immunoblotting against the myotube marker MHC. Lower panel, quantification of MHC levels 5 days after induction of differentiation. All data shown are mean ± S.D. ∗*p* < 0.05, ∗∗*p* < 0.01. MHC, myosin heavy chain; MyoD1, myoblast determination protein 1; TAZ-KO, TAFAZZIN-knockout.

**Supplemental Figure 3:**
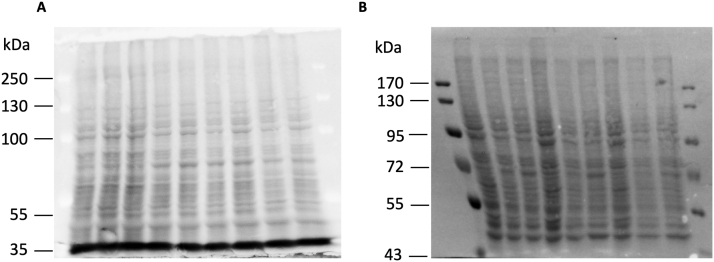


As expected from our mRNA data, MyoD1 protein was not detectable in EV-transfected TAZ-KO (TAZ-KO + EV) cells even after 7 days of differentiation induction. However, TAZ-KO cells transfected with the CMV-MyoD construct (TAZ-KO + MyoD1) showed MyoD1 expression comparable to WT controls (Figs. 3*A* and S3*A*), indicating that the MyoD1 construct is properly expressed in these cells.

After culturing WT + EV, TAZ-KO + EV, and TAZ-KO + MyoD1 cells for 5 or 7 days in differentiation medium (low serum medium to induce myoblast differentiation), we collected cells to perform immunoblotting for the myotube marker MHC. Strikingly, we found that MHC levels in TAZ-KO + MyoD1 cells were as high as in WT + EV controls, whereas MHC expression remained substantially reduced in the TAZ-KO + EV group (Figs. 3*B* and S3*B*). Together, our data strongly suggest that the myogenesis defects in TAZ-KO cells result from decreased MyoD1 and that these defects can be rescued by exogenous expression of MyoD1.

### TAZ-KO cells show increased MKX expression

MyoD1 can be transcriptionally repressed by MKX, a member of the Three Amino acid Loop Extension subfamily of homeodomain proteins (25, 26, 27). MKX can directly bind the *MyoD1* promoter, and interestingly, overexpression of MKX has been previously shown to inhibit MyoD1-induced conversion of 10T1/2 fibroblasts into myoblasts and differentiation of WT C2C12 myoblasts into myotubes, as evidenced by reduced levels of MyoD1 and MHC in these cells (28, 29).

**Figure 4:**
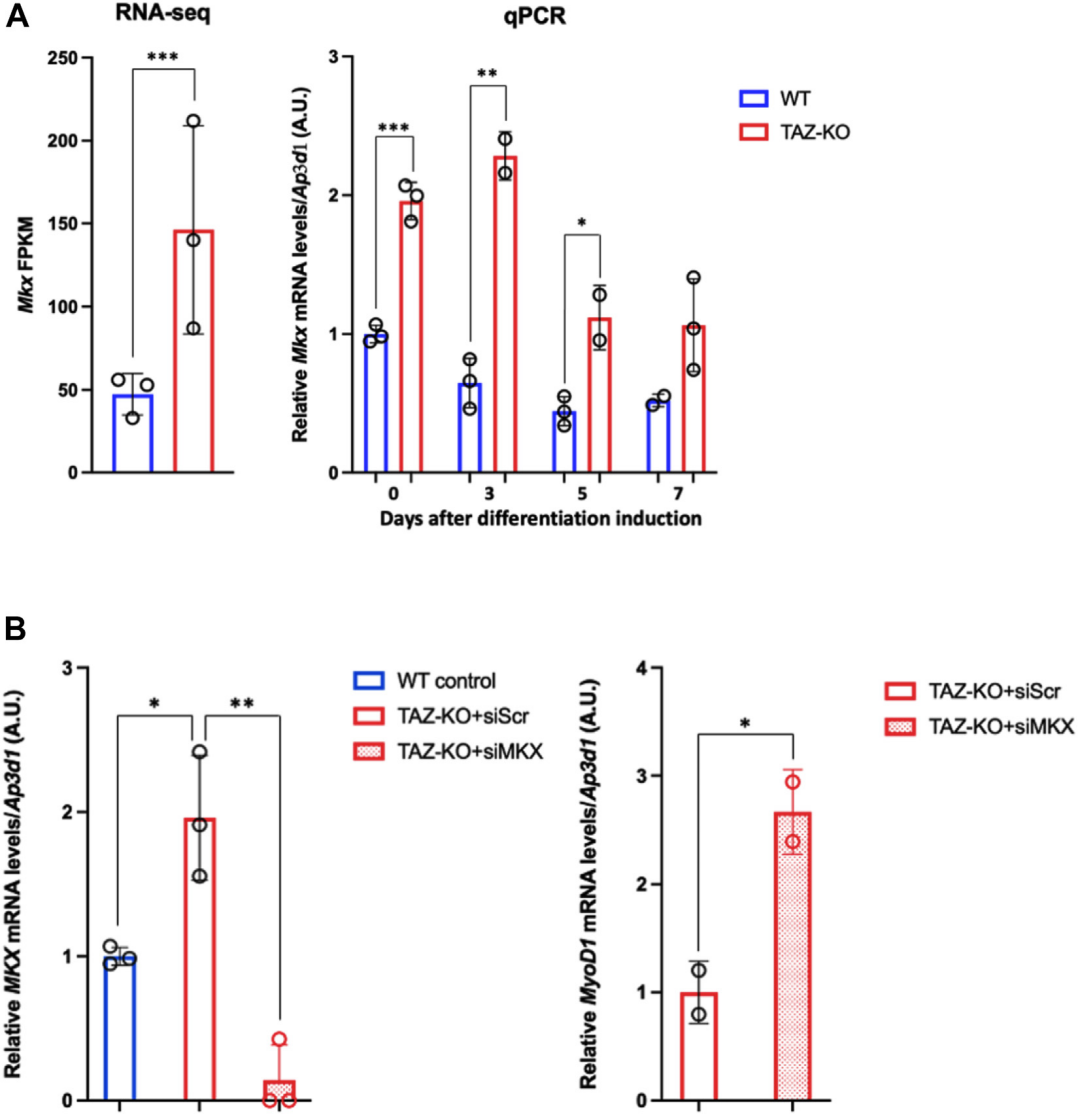
**Increased MyoD1 negative regulator MKX in TAZ-KO cells**. *A*, *Mkx* fragments per kilobase million (FPKM) values of WT and TAZ-KO myoblasts determined by RNA-seq (*left*), and qPCR analysis of *Mkx* mRNA levels in WT and TAZ-KO cells before and after differentiation induction (*right*). *Ap3d1* was used as an internal control. *B*, *left*: *Mkx* mRNA levels are significantly reduced in TAZ-KO cells transfected with MKX-targeting siRNAs compared to scrambled-treated cells. *Right*: *MyoD1* mRNA levels are increased in MKX siRNA-treated TAZ-KO cells compared to scrambled-treated cells. Data were obtained by qPCR analysis, and *Ap3d1* was used as an internal control. All data shown are mean ± S.D. ∗*p* < 0.05, ∗∗*p* <0.01, ∗∗∗*p* < 0.001. MKX, Mohawk; MyoD1, myoblast determination protein 1; qPCR, quantitative PCR; RNA-seq, RNA sequencing; TAZ-KO, TAFAZZIN-knockout.

To test the hypothesis that decreased MyoD1 levels are the result of elevated MKX expression, we evaluated levels of *Mkx* mRNA in WT and TAZ-KO cells. Consistent with our hypothesis, RNA-seq data showed that *Mkx* expression is 3-fold higher in TAZ-KO myoblasts compared to WT (Fig. 4*A*, left). *Mkx* mRNA levels also remained high during differentiation induction (Fig. 4*A*, right), suggesting that MyoD1 repression may results from persistent MKX up-regulation in the absence of TAFAZZIN. Intriguingly, when TAZ-KO cells were treated with MKX-targeted siRNAs to decrease MKX expression (Fig. 4*B*, left), MyoD1 expression was restored to levels in excess of WT cells (Fig. 4*B*, right). These data support a causative role for elevated MKX expression in the repression of MyoD1 in TAFAZZIN-deficient cells.

### Secretion of Wnt protein is decreased in TAZ-KO cells

**Figure 5:**
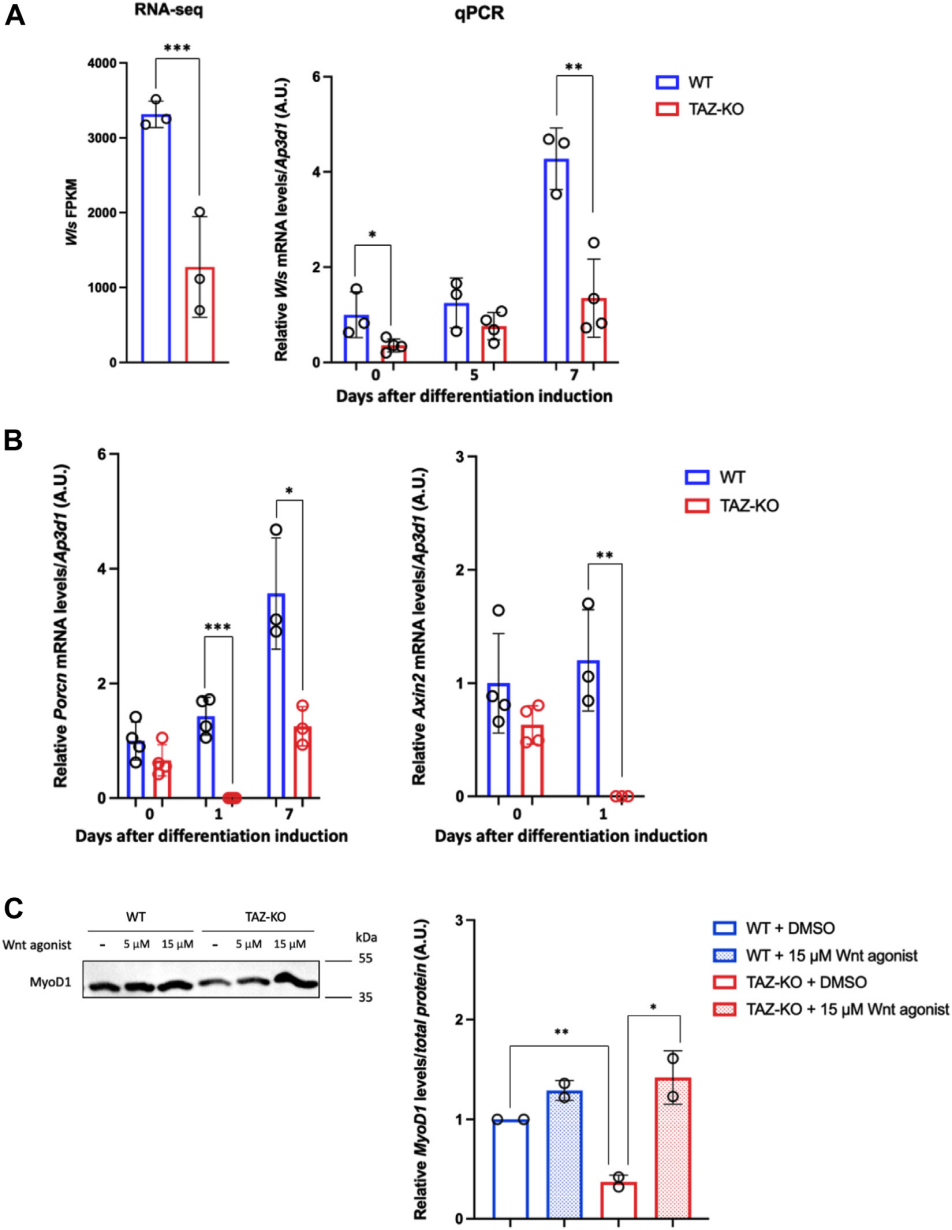
**Wnt signaling is decreased in TAZ-KO cells**. *A*, *Wls* mRNA levels determined by RNA-seq (*left*) and qPCR analysis (*right*) in WT and TAZ-KO cells before and after differentiation induction. *B*, qPCR analysis showed that *Porcn* (*left*) and *Axin2* (*right*) mRNA levels are decreased in TAZ-KO cells after 1 and 7 days of differentiation induction. *Ap3d1* was used as an internal control. *C*, *left*: MyoD1 protein levels are rescued by Wnt agonist treatment. After 5 days of differentiation induction, cells were treated with 5 μM or 10 μM Wnt agonist I in DMSO (CAS 853220–52–7, Calbiochem) for 48 h before subjecting to immunoblotting analysis against MyoD1. *Right*: quantification of MyoD1 protein levels as previously described. All data shown are mean ± S.D. ∗*p* < 0.05, ∗∗*p* < 0.01, ∗∗∗*p* <0.001. MyoD1, myoblast determination protein 1; qPCR, quantitative PCR; RNA-seq, RNA sequencing; TAZ-KO, TAFAZZIN-knockout; Wls, Wntless

The Wnt-triggered signal transduction pathway plays important roles in skeletal muscle development and tissue homeostasis (30), and because Wnt signaling has been shown to negatively regulate MKX expression, we predicted that Wnt signaling may also be decreased in TAZ-KO cells. Wnts are glycolipoproteins that are secreted into the extracellular environment in a process facilitated by the transmembrane packaging protein Wntless (Wls) (31, 32). Previous studies showed that depletion of Wls in both vertebrates and invertebrates results in Wnt loss-of-function phenotypes (33, 34, 35), indicating that the requirement for Wls to mediate Wnt signaling is fundamentally conserved across taxa. Expression of *Wls*, as determined by RNA-seq (Fig. 5*A*, left) and confirmed by qPCR analysis (Fig. 5*A*, right), was significantly lower in TAZ-KO cells compared to WT controls both before and after 7 days of differentiation induction. In addition, expression of Porcupine (Porcn), an acyltransferase that palmitoylates newly synthesized Wnt proteins and is required for Wnt secretion from ER to Golgi (32), was significantly decreased in TAZ-KO cells at 1 and 7 days post differentiation induction (Fig. 5*B*, left). Furthermore, expression of Axin2, a direct target of the Wnt pathway (36), was also lower in TAZ-KO cells after 1 day of differentiation induction (Fig. 5*B*, right). Together, our data suggest that loss of TAFAZZIN leads to decreased Wnt secretion and downstream signaling.

**Supplemental Figure 4:**
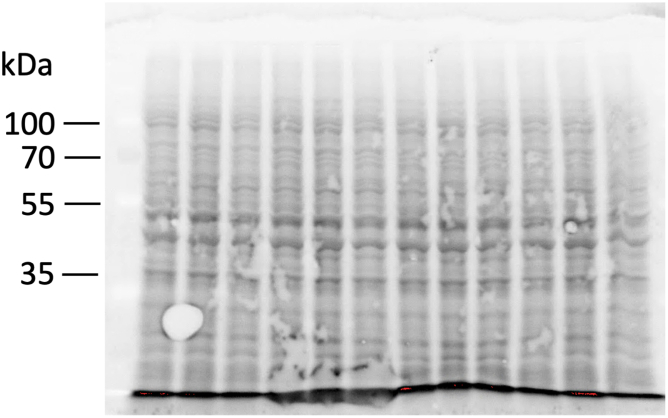


To test whether suppression of the Wnt-MKX regulatory cascade plays a causative role in repressing MyoD1, we treated WT and TAZ-KO cells with Wnt agonist I, a cell-permeable, selective activator of Wnt signaling (37). Strikingly, our data show that Wnt activation can restore MyoD1 expression in TAZ-KO cells to WT levels (Figs. 5*C* and S4), suggesting that Wnt activation can reverse MyoD1 repression in these cells.

## Discussion

Skeletal myopathy and muscle weakness are among the most debilitating phenotypes associated with BTHS. While it is widely agreed that skeletal myopathy likely results from mitochondrial dysfunction in the absence of TAFAZZIN, the mechanistic details have been largely unexplored. In this study, we determined that pathways responsible for cardiac and skeletal muscle development are highly decreased in the C2C12 BTHS myoblast model, TAZ-KO (Fig. 1). Importantly, we showed for the first time that the myoblast master transcription factor MyoD1 is highly repressed in TAZ-KO cells (Fig. 2, *A* and *B*) and in heart tissue from TAZ-KO mice, a BTHS mouse model (Fig. 2, *C* and *D*). These data suggest a potential mechanism that links TAFAZZIN deficiency with defective myogenesis and skeletal myopathy in BTHS. Our finding that exogenous MyoD1 can restore expression of MHC (Fig. 3), a marker of differentiated myoblasts, suggests that enhancing MyoD1 activity can rescue, at least in part, myogenesis defects caused by loss of TAFAZZIN.

It should be noted that in the current study, TAFAZZIN and the associated TAFAZZIN-knockout cell line “TAZ-KO” refer to a CL transacylase enzyme specifically implicated in BTHS pathophysiology. This enzyme is distinct from another protein referred to as “TAZ” (transcriptional coactivator with PDZ-binding motif) that has been implicated in MyoD-mediated myogenic differentiation through an unrelated mechanism (38).

Previous studies indicate that the canonical Wnt/β-catenin pathway is required for muscle-specific gene transcription during embryonic myogenesis and satellite cell-mediated muscle regeneration (39, 40). Specifically, Wnt/β-catenin is involved in switching myoblasts from a program of proliferation to myogenic differentiation (41), an outcome likely related to the negative regulatory relationship between Wnt signaling and MKX, a negative regulator of MyoD1 (25, 30). Therefore, it is tempting to speculate that deficient Wnt/β-catenin signaling may form the basis of the myogenesis defects in TAZ-KO cells. In this study, we present evidence of diminished Wnt signaling in TAZ-KO cells (Fig. 5), which is accompanied by elevated MKX expression (Fig. 4) and repression of MyoD1 (Fig. 2). Importantly, we also demonstrate that experimentally enhancing Wnt signaling rescues MyoD1 expression, suggesting that the Wnt-MKX regulatory cascade plays a causative role in suppressing myogenesis in TAFAZZIN-deficient cells.

Recent evidence suggests that there is robust bi-directional feedback between mitochondrial function and Wnt signaling activity (reviewed in (42)). On one hand, Wnt signaling modulates mitochondrial metabolism, as increased Wnt signaling activates mitochondrial biogenesis and increases mitochondria-derived reactive oxygen species generation (43), and Wnt3α overexpression increases both expression of mitochondrial genes and mitochondria number (44). On the other hand, mitochondrial retrograde signaling directly regulates Wnt pathways, showing bidirectional crosstalk between mitochondria and Wnt signaling. For instance, studies show that loss of mitochondrial DNA suppresses Wnt signaling (45) and mitochondrial stress-inducing agents (*e.g.*, oligomycin, FCCP, rotenone, antimycin A) also dampen Wnt signaling (46). Based on these findings, we propose that the mitochondrial dysfunction described in TAZ-deficient cells (18, 47, 48, 49) may act to suppress Wnt signaling, which leads to MKX upregulation and MyoD1 repression.

**Figure 6:**
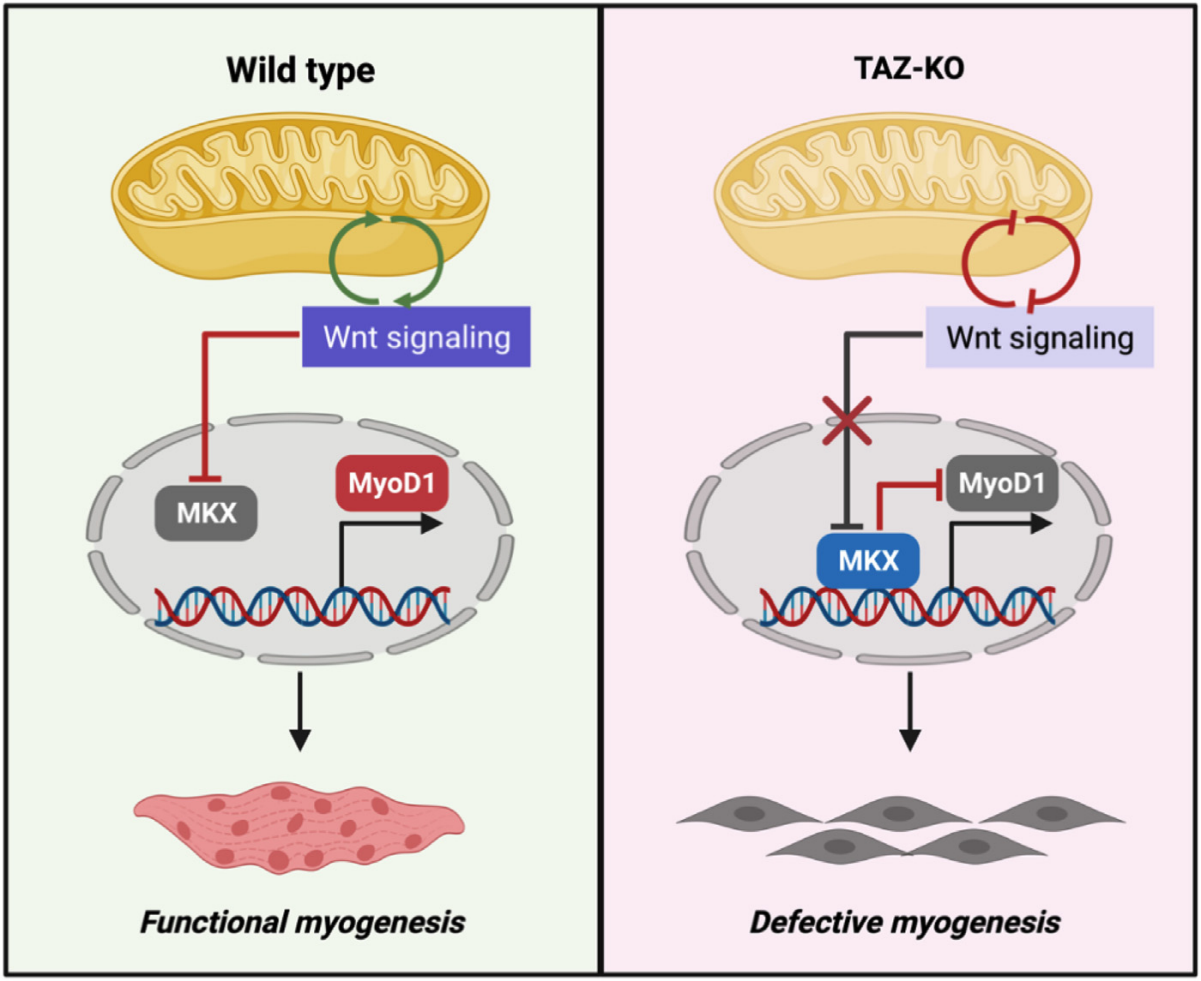
**Proposed mechanism of *MyoD1* repression in TAFAZZIN-deficient myoblasts**. Wnt signaling is an essential signal transduction pathway required for functional muscle development. Wnt signaling and mitochondrial function regulate one another through positive feedback. We propose that decreased mitochondrial function in the absence of TAFAZZIN leads to decreased Wnt signaling and a concomitant increase in MKX-mediated repression of MyoD1. MyoD1 is the master transcription factor that regulates muscle development and homeostasis. Therefore, repression of MyoD1 *via* the above mechanism may serve as a link between TAFAZZIN deficiency and skeletal myopathy in BTHS patients. *Image was created with**BioRender.com*. BTHS, Barth syndrome; MKX, Mohawk; MyoD1, myoblast determination protein 1.

Kishimoto *et al.* previously showed that activation of Wnt/β-catenin signaling strongly suppresses MKX (50), a direct negative regulator of MyoD1 transcription (28, 29). We hypothesize that in TAZ-KO cells, decreased Wnt signaling leads to upregulation of MKX, thus inhibiting MyoD1 transcription. Because MyoD1 is an important transcription factor and co-activator of several genes responsible for myocyte differentiation (12, 24), TAZ-KO cells with decreased MyoD1 levels exhibit myogenesis defects in response to myogenic stimuli (Fig. 6). Megeney *et al.* previously reported that loss of MyoD1 results in muscle regeneration defects in a Duchenne muscular dystrophy mouse model (51). Therefore, decreased MyoD1 in TAFAZZIN-deficient myoblasts may serve as the mechanism underlying the development of skeletal myopathy in BTHS patients.

It is important to point out that both the canonical (β-catenin-dependent) and noncanonical (β-catenin-independent) Wnt signaling pathways control the expression of myogenic regulatory factors, which are essential for myogenic lineage progression. Specifically, canonical Wnt signaling regulates the differentiation of muscle stem cells, whereas noncanonical signals mediate the self-renewal of satellite stem cells and the growth of muscle fibers (40). Our unpublished data indicate that β-catenin levels are not significantly different between WT and TAZ-KO cells (data not shown), suggesting that the noncanonical Wnt signaling pathway is responsible for dysfunctional MKX-MyoD1 regulation in TAFAZZIN-deficient cells. This is supported by work from Brunelli *et al.* showing that MyoD1 expression is dependent on noncanonical (*versus* canonical) Wnt signaling (52). However, future studies are needed to fully characterize the Wnt pathway players involved in this mechanism.

The current study represents the first transcriptomic analysis of the TAZ-KO mouse myoblast model of BTHS. We also show for the first time that MyoD1 is inhibited in TAZ-KO cells and propose a mechanism to explain how loss of TAFAZZIN leads to myogenesis defects. Although other groups have published transcriptomic studies in other BTHS models, including a mouse TAZ-KO neutrophil progenitor cell line (21) and TAFAZZIN-knockdown mouse hearts (53), MyoD1 was not identified as a significantly downregulated gene in these studies. This may be because these studies did not look specifically at myoblasts or skeletal myotubes, and according to the Mouse ENCODE Consortium data, MyoD1 expression is very limited in tissues outside of skeletal muscle, including neutrophils and heart tissue (54).

Little is known about the mechanisms underlying the pathology of skeletal myopathy in BTHS. The molecular mechanisms whereby CL, a phospholipid enriched in the mitochondria, regulates the complex program of muscle differentiation have not been previously studied. Our study suggests a new regulatory pathway that links CL with muscle development and thus identifies a potential new avenue for treating skeletal myopathy in BTHS patients by targeted elevation of MyoD1 expression. Future studies will be aimed at verifying the mechanistic details that link TAFAZZIN-mediated mitochondrial dysfunction and decreased Wnt signaling to the myogenic differentiation defects described here. Understanding this mechanism will no doubt contribute to our knowledge of the developmental defects in BTHS as well as other mitochondrial diseases.

## Experimental procedures

### Cell line and growth conditions

The TAZ-KO C2C12 cell line was generated using CRISPR-Cas9 as previously described (18, 19). Growth medium consists of Dulbecco's modified Eagle's medium (Gibco) containing 10% FBS (Atlanta), 2 mM glutamine (Gibco), penicillin (100 units/ml), and streptomycin (100 μg/ml) (both from Invitrogen Corp). Cells were grown at 37 °C in a humidified incubator with 5% CO_2_. To induce myoblast differentiation, WT and TAZ-KO C2C12 cells reaching 80% confluency were switched to Dulbecco's modified Eagle's medium (Gibco) containing 2% horse serum (Gibco), 2 mM glutamine (Gibco), penicillin (100 units/ml), and streptomycin (100 μg/ml) (both from Invitrogen Corp) until the indicated time points. Fresh medium for differentiation induction was changed every 24 h.

### Animals and heart tissue collection

TAZ-KO mice were described previously (55). Adult (9–15 months of age) male TAZ-KO mice and their male wildtype littermates were sacrificed by CO_2_ narcosis and cervical dislocation. Heart tissues (ventricle) were dissected, rinsed in PBS, snap frozen in liquid nitrogen, and stored in a −80 °C freezer for later use. To isolate total protein and RNA, 30 to 40 mg of tissue was homogenized by sonication in RIPA buffer on ice. After centrifugation, the supernatants containing protein were used for immunoblotting analysis, whereas tissue pellets were used to extract total RNA using the RNeasy Plus Mini Kit (QIAGEN).

## RNA-seq analysis

### RNA isolation

Total RNA was extracted from undifferentiated TAZ-KO and isogenic WT C2C12 cells using TRIzol reagent (Invitrogen Corp) following the manufacturer’s instructions.

### 3′ mRNA expression analysis

Expression analysis was conducted by the Wayne State University Genome Sciences Core as previously described (56). Three biological replicates were used for each condition. QuantSeq 3′ mRNA-Seq Library Prep Kit FWD for Illumina (Lexogen) was used to generate libraries of sequences close to the 3′ end of polyadenylated RNA from 15 ng of total RNA in 5 μl of nuclease-free water following a low-input protocol. Library aliquots were assessed for overall quality using ScreenTape for the Agilent 2200 TapeStation and quantified using Qubit 1× dsDNA HS Assay kit (Invitrogen Corp). Barcoded libraries were normalized to 2 nM before sequencing at 300 PM on one lane of a NovaSeq 6000 SP flow cell. After de-multiplexing with Illumina's CASAVA 1.8.2 software, the 75 bp reads were aligned to the mouse genome (Build hg38) with STAR_2.4 (57) and tabulated for each gene region (58). Differential gene expression analysis was used to compare transcriptome changes between conditions using edgeR v.3.22.3 (59). Genes differentially expressed with a fold change of >1.5 and adjusted *p*-value of <0.05 were considered significant. The top 100 downregulated genes based on their fold changes were analyzed and grouped by their biological functions using ClueGO (20). Significantly changed genes were analyzed by PANTHER pathway analysis (22, 23) with cutoffs at a fold change >2 and *p*-value < 0.05.

### Transient expression of MyoD1 in C2C12 cells

Exogenous mouse MyoD1 was expressed in TAZ-KO myoblasts by transfecting C2C12 cells with a CMV-MyoD plasmid using Lipofectamine 3000 (Thermo Fisher) according to the manufacturer’s instructions. CMV-MyoD was a gift from Andrew Lassar (Addgene plasmid #8398; http://n2t.net/addgene:8398; RRID: Addgene_8398) (60). The EV pCS2+8CeGFP was a gift from Amro Hamdoun (Addgene plasmid #34952; http://n2t.net/addgene:34952, RRID:Addgene_34952) (61). Briefly, 7.5 to 15 × 10^4^ cells were plated into each well of a 6-well tissue culture plate. On the following day, cells were transfected with 2.5 μg CMV-MyoD plasmid or EV control. The DNA-Lipofectamine mix was incubated at room temperature for 30 min. After transfection, the medium was replaced with differentiation medium (containing 2% horse serum) containing antibiotics.

### Knockdown of MKX by siRNAs

siRNAs targeting mouse MKX and universal scrambled negative control siRNA were purchased from OriGene (Catalog No SR411975). siRNAs (25 nM) were transfected into C2C12 cells grown in 6- or 12-well dishes to approximately 80% confluency using the *Trans*IT-X2 Dynamic Delivery System (Mirus Bio) according to the manufacturer’s instructions. Seventy-two hours post transfection, knockdown efficiency was monitored by qPCR analysis.

### Immunoblotting

Protein concentration from total cell extract was determined using the DC Protein Assay Kit (BIO-RAD). Protein lysate was analyzed on an 11% (for MyoD1) or 8% (for MHC) SDS-PAGE gel. Immunoblotting was performed using primary antibodies against MHC (R&D Systems; catalog number MAB4470-SP; 1:200 dilution) and MyoD1 (abcam; catalog number ab64159; 1:1000 dilution). All primary antibody incubations were performed overnight at 4 °C on a 360° rotator. Corresponding secondary antibodies conjugated to europium (Molecular Devices; 1:10,000) or horseradish peroxidase (Thermo; 1:10,000) were used to detect immunoreactivity. Enhanced chemiluminescence substrate (Thermo) was used to visualize the signal of horseradish peroxidase.

### Real-time quantitative PCR

The iScript cDNA Synthesis Kit (BIO-RAD) was used to synthesize complementary DNA from the indicated samples. Real-time qPCR analysis was performed using PowerUp SYBR Green Master Mix (Thermo). qPCRs were carried out in a QuantStudio 3 Real-Time PCR system (Thermo). *ACTB* or *Ap3d1* was used as an internal control to normalize levels of mRNAs. *Ap3d1* was used to normalize gene expression of C2C12 cells during myogenesis as previously discussed in (62). Primers used for amplification are listed below.

**Table:** 

Primer	Sequence
*MyoD1* forward	CCACTCCGGGACATAGACTTG
*MyoD1* reverse	AAAAGCGCAGGTCTGGTGAG
*Mkx* forward	GCAGAATGGAGGGAAGGTAAG
*Mkx* reverse	GGTTGTCACGGTGCTTGTA
*ACTB* forward	GGTCGTACCACAGGCATTGTGATG
*ACTB* reverse	GGAGAGCATAGGCCTCGTAGATGG
*Ap3d1* forward	TCGACCGCATGTTCGATAAG
*Ap3d1* reverse	GTCAATGCACTGGGAGATGTA
*Wls* forward	ACTGCAGCTTACTACCACTATAAC
*Wls* reverse	GCCATACCATAGCCTCCTATTC
*Porcn* forward	GTCCCTGGCATTCATCACTTA
*Porcn* reverse	CAGACAACGCTTTGACAAGATG
*Axin2* forward	GATGTCTGGCAGTGGATGTTAG
*Axin2* reverse	GACTCCAATGGGTAGCTCTTTC

## Data availability

All data are available in the main text or the supplemental materials.

## Supporting information

This article contains supporting information.

## Conflict of interest

The authors declare no conflict of interest with the contents of this article.

## References

[1] Bione S., D'Adamo P., Maestrini E., Gedeon A.K., Bolhuis P.A., Toniolo D. (1996). A novel X-linked gene, G4.5. is responsible for Barth syndrome. Nat. Genet..

[2] Vreken P., Valianpour F., Nijtmans L.G., Grivell L.A., Plecko B., Wanders R.J. (2000). Defective remodeling of cardiolipin and phosphatidylglycerol in Barth syndrome. Biochem. Biophys. Res. Commun..

[3] Claypool S.M., Koehler C.M. (2012). The complexity of cardiolipin in health and disease. Trends Biochem. Sci..

[4] Schlame M., Greenberg M.L. (2017). Biosynthesis, remodeling and turnover of mitochondrial cardiolipin. Biochim. Biophys. Acta Mol. Cell Biol. Lipids.

[5] Shen Z., Ye C., Mccain K., Greenberg M.L. (2015). The role of cardiolipin in cardiovascular health. Biomed. Res. Int..

[6] Ye C., Shen Z., Greenberg M.L. (2016). Cardiolipin remodeling: a regulatory hub for modulating cardiolipin metabolism and function. J. Bioenerg. Biomembr..

[7] Barth P.G., Scholte H.R., Berden J.A., Van der Klei-Van Moorsel J.M., Luyt-Houwen I.E., Van 't Veer-Korthof E.T. (1983). An X-linked mitochondrial disease affecting cardiac muscle, skeletal muscle and neutrophil leucocytes. J. Neurol. Sci..

[8] Kelley R.I., Cheatham J.P., Clark B.J., Nigro M.A., Powell B.R., Sherwood G.W. (1991). X-linked dilated cardiomyopathy with neutropenia, growth retardation, and 3-methylglutaconic aciduria. J. Pediatr..

[9] Bittel A.J., Bohnert K.L., Reeds D.N., Peterson L.R., de Las fuentes L., Corti M. (2018). Reduced muscle strength in Barth syndrome may be improved by resistance exercise training: a pilot study. JIMD Rep..

[10] Mazar I., Stokes J., Ollis S., Love E., Espensen A., Barth P.G. (2019). Understanding the life experience of Barth syndrome from the perspective of adults: a qualitative one-on-one interview study. Orphanet J. Rare Dis..

[11] Schmidt M., Schuler S.C., Huttner S.S., von Eyss B., von Maltzahn J. (2019). Adult stem cells at work: regenerating skeletal muscle. Cell Mol. Life Sci..

[12] Davis R.L., Weintraub H., Lassar A.B. (1987). Expression of a single transfected cDNA converts fibroblasts to myoblasts. Cell.

[13] Weintraub H., Tapscott S.J., Davis R.L., Thayer M.J., Adam M.A., Lassar A.B. (1989). Activation of muscle-specific genes in pigment, nerve, fat, liver, and fibroblast cell lines by forced expression of MyoD. Proc. Natl. Acad. Sci. U. S. A..

[14] de la Serna I.L., Roy K., Carlson K.A., Imbalzano A.N. (2001). MyoD can induce cell cycle arrest but not muscle differentiation in the presence of dominant negative SWI/SNF chromatin remodeling enzymes. J. Biol. Chem..

[15] Megeney L.A., Kablar B., Garrett K., Anderson J.E., Rudnicki M.A. (1996). MyoD is required for myogenic stem cell function in adult skeletal muscle. Genes Dev..

[16] Puri P.L., Avantaggiati M.L., Balsano C., Sang N., Graessmann A., Giordano A. (1997). p300 is required for MyoD-dependent cell cycle arrest and muscle-specific gene transcription. EMBO J..

[17] Thorburn A.M., Walton P.A., Feramisco J.R. (1993). MyoD induced cell cycle arrest is associated with increased nuclear affinity of the Rb protein. Mol. Biol. Cell.

[18] Li Y., Lou W., Raja V., Denis S., Yu W., Schmidtke M.W. (2019). Cardiolipin-induced activation of pyruvate dehydrogenase links mitochondrial lipid biosynthesis to TCA cycle function. J. Biol. Chem..

[19] Lou W., Reynolds C.A., Li Y., Liu J., Huttemann M., Schlame M. (2018). Loss of tafazzin results in decreased myoblast differentiation in C2C12 cells: a myoblast model of Barth syndrome and cardiolipin deficiency. Biochim. Biophys. Acta Mol. Cell Biol. Lipids.

[20] Bindea G., Mlecnik B., Hackl H., Charoentong P., Tosolini M., Kirilovsky A. (2009). ClueGO: a cytoscape plug-in to decipher functionally grouped gene ontology and pathway annotation networks. Bioinformatics.

[21] Sohn J., Milosevic J., Brouse T., Aziz N., Elkhoury J., Wang S. (2022). A new murine model of Barth Syndrome neutropenia links TAFAZZIN deficiency to increased ER stress induced apoptosis. Blood Adv..

[22] Mi H., Ebert D., Muruganujan A., Mills C., Albou L.P., Mushayamaha T. (2021). PANTHER version 16: a revised family classification, tree-based classification tool, enhancer regions and extensive API. Nucl. Acids Res..

[23] Mi H., Thomas P. (2009). PANTHER pathway: an ontology-based pathway database coupled with data analysis tools. Met. Mol. Biol..

[24] Tapscott S.J. (2005). The circuitry of a master switch: myod and the regulation of skeletal muscle gene transcription. Development.

[25] Bilioni A., Craig G., Hill C., Mcneill H. (2005). Iroquois transcription factors recognize a unique motif to mediate transcriptional repression *in vivo*. Proc. Natl. Acad. Sci. U. S. A..

[26] Gomez-Skarmeta J.L., Modolell J. (2002). Iroquois genes: genomic organization and function in vertebrate neural development. Curr. Opin. Genet. Dev..

[27] Wang G.F., Nikovits W., Bao Z.Z., Stockdale F.E. (2001). Irx4 forms an inhibitory complex with the vitamin D and retinoic X receptors to regulate cardiac chamber-specific slow MyHC3 expression. J. Biol. Chem..

[28] Anderson D.M., Beres B.J., Wilson-Rawls J., Rawls A. (2009). The homeobox gene Mohawk represses transcription by recruiting the sin3A/HDAC co-repressor complex. Dev. Dyn..

[29] Chuang H.N., Hsiao K.M., Chang H.Y., Wu C.C., Pan H. (2014). The homeobox transcription factor Irxl1 negatively regulates MyoD expression and myoblast differentiation. FEBS J..

[30] van Amerongen R., Nusse R. (2009). Towards an integrated view of Wnt signaling in development. Development.

[31] Das S., Yu S., Sakamori R., Stypulkowski E., Gao N. (2012). Wntless in Wnt secretion: molecular, cellular and genetic aspects. Front. Biol. (Beijing).

[32] Mehta S., Hingole S., Chaudhary V. (2021). The emerging mechanisms of Wnt secretion and signaling in development. Front. Cell Dev. Biol..

[33] Carpenter A.C., Rao S., Wells J.M., Campbell K., Lang R.A. (2010). Generation of mice with a conditional null allele for Wntless. Genesis.

[34] Fu J., Ivy Yu H.M., Maruyama T., Mirando A.J., Hsu W. (2011). Gpr177/mouse Wntless is essential for Wnt-mediated craniofacial and brain development. Dev. Dyn..

[35] Silhankova M., Port F., Harterink M., Basler K., Korswagen H.C. (2010). Wnt signalling requires MTM-6 and MTM-9 myotubularin lipid-phosphatase function in Wnt-producing cells. EMBO J..

[36] Jho E.H., Zhang T., Domon C., Joo C.K., Freund J.N., Costantini F. (2002). Wnt/beta-catenin/Tcf signaling induces the transcription of Axin2, a negative regulator of the signaling pathway. Mol. Cell Biol..

[37] Liu J., Wu X., Mitchell B., Kintner C., Ding S., Schultz P.G. (2005). A small-molecule agonist of the Wnt signaling pathway. Angew. Chem. Int. Ed. Engl..

[38] Jeong H., Bae S., An S.Y., Byun M.R., Hwang J.H., Yaffe M.B. (2010). TAZ as a novel enhancer of MyoD-mediated myogenic differentiation. FASEB J..

[39] Hwang Y., Suk S., Shih Y.R., Seo T., Du B., Xie Y. (2014). WNT3A promotes myogenesis of human embryonic stem cells and enhances *in vivo* engraftment. Sci. Rep..

[40] von Maltzahn J., Chang N.C., Bentzinger C.F., Rudnicki M.A. (2012). Wnt signaling in myogenesis. Trends Cell Biol..

[41] Tanaka S., Terada K., Nohno T. (2011). Canonical Wnt signaling is involved in switching from cell proliferation to myogenic differentiation of mouse myoblast cells. J. Mol. Signal..

[42] Delgado-Deida Y., Alula K.M., Theiss A.L. (2020). The influence of mitochondrial-directed regulation of Wnt signaling on tumorigenesis. Gastroenterol. Rep. (Oxf).

[43] Yoon J.C., Ng A., Kim B.H., Bianco A., Xavier R.J., Elledge S.J. (2010). Wnt signaling regulates mitochondrial physiology and insulin sensitivity. Genes Dev..

[44] Ning X., He J., Shi X., Yu T., Yang G. (2019). Wnt3a regulates mitochondrial biogenesis through p38/CREB pathway. Biochem. Biophys. Res. Commun..

[45] Wen Y.A., Xiong X., Scott T., Li A.T., Wang C., Weiss H.L. (2019). The mitochondrial retrograde signaling regulates Wnt signaling to promote tumorigenesis in colon cancer. Cell Death Differ..

[46] Costa R., Peruzzo R., Bachmann M., Monta G.D., Vicario M., Santinon G. (2019). Impaired mitochondrial ATP production downregulates Wnt signaling *via* ER stress induction. Cell Rep..

[47] Li Y., Lou W., Grevel A., Bottinger L., Liang Z., Ji J. (2020). Cardiolipin-deficient cells have decreased levels of the iron-sulfur biogenesis protein frataxin. J. Biol. Chem..

[48] Raja V., Joshi A.S., Li G., Maddipati K.R., Greenberg M.L. (2017). Loss of cardiolipin leads to perturbation of acetyl-CoA Synthesis. J. Biol. Chem..

[49] Raja V., Salsaa M., Joshi A.S., Li Y., van Roermund C.W.T., Saadat N. (2019). Cardiolipin-deficient cells depend on anaplerotic pathways to ameliorate defective TCA cycle function. Biochim. Biophys. Acta Mol. Cell Biol. Lipids.

[50] Kishimoto Y., Ohkawara B., Sakai T., Ito M., Masuda A., Ishiguro N. (2017). Wnt/beta-catenin signaling suppresses expressions of Scx, Mkx, and Tnmd in tendon-derived cells. PLoS One.

[51] Megeney L.A., Kablar B., Perry R.L., Ying C., May L., Rudnicki M.A. (1999). Severe cardiomyopathy in mice lacking dystrophin and MyoD. Proc. Natl. Acad. Sci. U. S. A..

[52] Brunelli S., Relaix F., Baesso S., Buckingham M., Cossu G. (2007). Beta catenin-independent activation of MyoD in presomitic mesoderm requires PKC and depends on Pax3 transcriptional activity. Dev. Biol..

[53] Schafer C., Moore V., Dasgupta N., Javadov S., James J.F., Glukhov A.I. (2018). The effects of PPAR stimulation on cardiac metabolic pathways in Barth syndrome mice. Front. Pharmacol..

[54] Yue F., Cheng Y., Breschi A., Vierstra J., Wu W., Ryba T. (2014). A comparative encyclopedia of DNA elements in the mouse genome. Nature.

[55] Ren M., Xu Y., Erdjument-Bromage H., Donelian A., Phoon C.K.L., Terada N. (2019). Extramitochondrial cardiolipin suggests a novel function of mitochondria in spermatogenesis. J. Cell Biol..

[56] Shah E.J., Gurdziel K., Ruden D.M. (2020). Drosophila exhibit divergent sex-based responses in transcription and motor function after traumatic brain injury. Front. Neurol..

[57] Dobin A., Davis C.A., Schlesinger F., Drenkow J., Zaleski C., Jha S. (2013). Star: ultrafast universal RNA-seq aligner. Bioinformatics.

[58] Anders S., Pyl P.T., Huber W. (2015). HTSeq--a Python framework to work with high-throughput sequencing data. Bioinformatics.

[59] Robinson M.D., Mccarthy D.J., Smyth G.K. (2010). edgeR: a Bioconductor package for differential expression analysis of digital gene expression data. Bioinformatics.

[60] Skapek S.X., Rhee J., Kim P.S., Novitch B.G., Lassar A.B. (1996). Cyclin-mediated inhibition of muscle gene expression *via* a mechanism that is independent of pRB hyperphosphorylation. Mol. Cell Biol..

[61] Gokirmak T., Campanale J.P., Shipp L.E., Moy G.W., Tao H., Hamdoun A. (2012). Localization and substrate selectivity of sea urchin multidrug (MDR) efflux transporters. J. Biol. Chem..

[62] Hildyard J.C., Wells D.J. (2014). Identification and validation of quantitative PCR reference genes suitable for normalizing expression in normal and dystrophic cell culture models of myogenesis. PLoS Curr..

